# Expression of Tgfβ1 and Inflammatory Markers in the 6-hydroxydopamine Mouse Model of Parkinson’s Disease

**DOI:** 10.3389/fnmol.2016.00007

**Published:** 2016-02-03

**Authors:** Stefan Jean-Pierre Haas, Xiaolai Zhou, Venissa Machado, Andreas Wree, Kerstin Krieglstein, Björn Spittau

**Affiliations:** ^1^Institute of Anatomy, Rostock University Medical CenterRostock, Germany; ^2^Department of Molecular Embryology, Institute of Anatomy and Cell Biology, Albert-Ludwigs-UniversityFreiburg, Germany; ^3^Department of Molecular Biology and Genetics, Weill Institute for Cell and Molecular Biology, Cornell UniversityIthaca, NY, USA; ^4^Spemann Graduate School of Biology and Medicine (SGBM), Albert-Ludwigs-UniversityFreiburg, Germany; ^5^Faculty of Biology, Albert-Ludwigs-UniversityFreiburg, Germany

**Keywords:** 6-OHDA, microglia, astrocytes, Tnfα, Tgfβ1

## Abstract

Parkinson’s disease (PD) is a neurodegenerative disorder that is characterized by loss of midbrain dopaminergic (mDA) neurons in the substantia nigra (SN). Microglia-mediated neuroinflammation has been described as a common hallmark of PD and is believed to further trigger the progression of neurodegenerative events. Injections of 6-hydroxydopamine (6-OHDA) are widely used to induce degeneration of mDA neurons in rodents as an attempt to mimic PD and to study neurodegeneration, neuroinflammation as well as potential therapeutic approaches. In the present study, we addressed microglia and astroglia reactivity in the SN and the caudatoputamen (CPu) after 6-OHDA injections into the medial forebrain bundle (MFB), and further analyzed the temporal and spatial expression patterns of pro-inflammatory and anti-inflammatory markers in this mouse model of PD. We provide evidence that activated microglia as well as neurons in the lesioned SN and CPu express Transforming growth factor β1 (Tgfβ1), which overlaps with the downregulation of pro-inflammatory markers *Tnfα*, and *iNos*, and upregulation of anti-inflammatory markers *Ym1* and *Arg1*. Taken together, the data presented in this study suggest an important role for Tgfβ1 as a lesion-associated factor that might be involved in regulating microglia activation states in the 6-OHDA mouse model of PD in order to prevent degeneration of uninjured neurons by microglia-mediated release of neurotoxic factors such as Tnfα and nitric oxide (NO).

## Introduction

Parkinson’s disease (PD) is a neurodegenerative disorder that is characterized by loss of midbrain dopaminergic (mDA) neurons in the substantia nigra (SN) and the subsequent loss of axonal projections to the caudatoputamen (CPu) resulting in decreased dopamine levels (Jellinger, 2001). Although the causes that trigger disease onset remain elusive in the majority of cases and environmental factors are discussed (Goldman, 2014), neuroinflammation mediated by microglia has been described as a common hallmark of PD and is believed to fuel the progression of neurodegenerative events by releasing neurotoxic factors such as Tnfα and nitric oxide (NO; Block et al., 2007; Hirsch and Hunot, 2009). Microglia—the resident immune cells of the central nervous system (CNS)—are involved in a plethora of neurodegenerative pathologies (Prinz and Priller, 2014) and interestingly, show a higher density in the SN and CPu as compared to other brain areas (Lawson et al., 1990; Sharaf et al., 2013). In human PD cases, microglia reactivity has been extensively described (McGeer and McGeer, 2008). Moreover, due to their unique origin (Prinz and Priller, 2014), microglia undergo ageing and are believed to become senescent, a phenomenon which is likely to impair their normal functions (Streit, 2006). Interestingly, aged microglia show a propensity towards inflammatory reaction states (Lee et al., 2013) indicating that ageing might generate an environment that could be harmful for mDA neurons and potentially explain the age-dependent onset of PD.

Experimental approaches to develop sufficient animal models for PD, which reproduce features of the human disease, have not yet resulted in a successful model. Thus, toxin-based animal models using 1-methyl-4-phenyl-1,2,3,6-tetrahydropyridine (MPTP) and 6-hydroxydopamine (6-OHDA) are widely used to mimic the PD situation in rodents and to study neurodegeneration, neuroinflammation as well as potential therapeutic approaches (Schober, 2004). Injection of 6-OHDA has been shown to result in a rapid decrease in tyrosine hydroxylase (TH)^+^ fiber densities in the CPu and a delayed apoptotic cell death of TH^+^ dopaminergic neurons in the midbrain which follows several days after initial injection of the toxin (Martí et al., 2002; Stott and Barker, 2014). Notably, activation of microglia occurs rapid after injection of 6-OHDA and has been demonstrated to precede the degeneration of mDA neurons (Marinova-Mutafchieva et al., 2009; Walsh et al., 2011). 6-OHDA-intoxicated neurons die by apoptotic cell death (Martí et al., 2002), which normally would not result in an inflammatory response. However, either 6-OHDA-induced neuronal impairment, or released inflammatory factors result in microglia activation prior to neuron death. Moreover, direct physical interactions and cell-to-cell communications between microglia and neurons are essential during 6-OHDA-induced neurodegeneration and neuroinflammation (Virgone-Carlotta et al., 2013). Due to the fact that microglia-mediated neuroinflammation is able to increase and accelerate progression of mDA neurodegeneration (Block et al., 2007), the extent and duration of microglia activation has to be tightly regulated in order to prevent threatening of healthy uninjured neurons. One of the most potent endogenous factors regulating microglia activation is Transforming growth factor β1 (Tgfβ1), which has been shown to be essential for microglia homeostasis *in vivo* (Butovsky et al., 2014) and *in vitro* (Spittau et al., 2013). Moreover, Tgfβ1 sufficiently blocks microglia activation induced by LPS (Kim et al., 2004) and IFNγ (Herrera-Molina et al., 2012; Zhou et al., 2015) and is further important for the adoption of a microglia alternative activation phenotype, which is triggered by interleukin 4 (IL4) and is believed to promote tissue repair and neuroprotection (Zhou et al., 2012).

In the present study, we describe microglia and astroglia reactivity in the SN and CPu after 6-OHDA injection into the medial forebrain bundle (MFB), and further demonstrate the temporal and spatial expression patterns of pro-inflammatory and anti-inflammatory markers in this mouse model of PD. Finally, we provide evidence that activated microglia as well as neurons in the lesioned SN and CPu express Tgfβ1, which precedes the downregulation of pro-inflammatory markers. Together, our data suggest an important role for Tgfβ1 as a lesion-associated factor that might regulate expression of inflammatory markers in the 6-OHDA model of PD.

## Materials and Methods

### Animals

Male C57BL/6-mice weighting about 22 g at the beginning of the experiments were housed at 22 ± 2°C under a 12 h light/dark cycle with free access to food and water. All animal-related procedures were conducted in accordance with the local ethical guidelines and have been approved by the animal experimentation committee of the University of Rostock.

### Generation of Hemiparkinsonian Mice

Mice (*n* = 42) were deeply anaesthetized by an intraperitoneal injection of Ketamin (75 mg/kg) and Rompun (5.8 mg/kg) and mounted in a mouse adaptor (Stoelting Co) fixed in a rat stereotaxic apparatus (Kopf, Tujunga, CA, USA). The skull was opened with a dental thrill and animals were unilaterally lesioned by an injection of 6-hydroxydopamine (6-OHDA-HCl, Sigma) into the right MFB via a 26 ga Hamilton syringe [5 μg 6-OHDA/2 μl, solved in 0.9% saline containing 0.02% ascorbic acid (Merck), coordinates referring to bregma: AP −1.2, ML −1.1, V −5 (Dura)] (Paxinos and Franklin, 2011). The solution was injected over a time period of 4 min, after further 3 min the syringe was pulled of slowly and the skin was sutured. Then the animals received supplementary baby food (cereals and fruit mash) and chocolate to prevent heavy weight loss.

### Tissue Processing and Immunohistochemistry

Mice were injected with an overdose of pentobarbital (60 mg/kg) and transcardially perfused with ice cold 0.9% sodium chloride (10 ml), followed by 50 ml of 3.7% PFA. Brains were immediately removed from the skull, postfixed overnight, and transferred into 0.1 M PBS containing 20% sucrose (overnight, 4°C). The cryoprotected brains were frozen in isopentane (−50°C) and stored at −80°C. Brains were cut with a cryostat at 30 μm and serial sections were collected free-floating in a cryoprotective solution and stored at −20°C until further processing. For immunohistochemical analysis of neuronal loss, microgliosis and astrogliosis, sections from SN and caudatoputamen (CPu) were washed three times with PBS, blocked with 10% normal goat serum (Vector Laboratories) and 0.1% Triton-X/100 for 1 h at room temperature (RT), and then incubated with anti-TH (1:500, polyclonal, Millipore), anti-Iba1 (1:500, polyclonal, Wako), anti-Gfap (1:800, monoclonal, Millipore), anti-Map2 (1:500, polyclonal, Abcam) primary antibodies overnight at 4°C. This was followed by incubation with goat anti-mouse or goat anti-rabbit fluorescence-coupled secondary antibodies (1:200, Cell Signaling Technologies) for 1 h at RT. The sections were washed three times with PBS for 3 min each and nuclei were counterstained using 4′,6-diamidino-2-phenylindole (DAPI, Roche). After final washing, sections were placed on objective slides and mounted with Fluoromount medium (SouthernBiotech). Fluorescence images were captured using the Leica AF6000 imaging system (LEICA, Wetzlar, Germany) and the Zeiss AxioImager M2 (Zeiss, Göttingen, Germany).

### RNA Isolation and Reverse Transcription

Mice were sacrificed by cervical dislocation at the designated timepoints and the SN and CPu from the lesioned and the unlesioned hemispheres were dissected and transferred to RNA later (Ambion). The tissues were then homogenized in peqGOLD TriFast (Peqlab) using the Precellys 24 homogenizer (Peqlab). RNA was extracted according to the manufacturer’s instructions. Quality and concentration of isolated RNA was determined using a NanoDrop spectrophotometer (Thermo Scientific). 1 μg RNA was reverse transcribed to cDNA using the GeneAmp RNA PCR Core Kit (Applied Biosystems) and random hexamer primers according to manufacturer’s instructions.

### Quantitative RT-PCR

Expression of inflammatory and anti-inflammatory factors was detected using cDNAs from control and lesioned SN and CPu isolated at 1 day, 2 days, 6 days, 8 days and 14 days after 6-OHDA injections into the MFB. Quantitative RT-PCR analysis was performed using the MyiQ^TM^ (BIO-RAD, München, Germany) and the Quantitect SYBR Green PCR Kit (Applied Biosystems, Darmstadt, Germany) with 1 μl of cDNA template in a 25 μl reaction mixture. Results were analyzed using the Bio-Rad iQ5 Opitcal System Software and the comparative CT method. Data throughout the study are expressed as 2^−ΔΔCT^ for the experimental gene of interest normalized to the housekeeping gene *Gapdh* and presented as fold change relative to the unlesioned control side of each individual mouse. The primers used throughout this study are: iNos*for* 5′-TTGACGCTCGGAACTGTAGCAC-3′, iNos*rev* 5′-CGACCTGATGTTGCCATTGTTG-3′ [NM_010927], Tnfα*for* 5′-TCTACTGAACTTCGGGGTGATCG-3′, Tnfα*rev* 5′-TGATCTGAGTGTGAGGGTCTGGG-3′ [NM_013693.3], Ym1*for* 5′-AGACTTGCGTGACTATGAAGCATTG-3′, Ym1*rev* 5′-GCAGGTCCAAACTTACATCCTC-3′ [NM_009892.2], Arg1*for* 5′-TCATGGAAGTGAACCCAACTCTTG-3′, Arg1*rev* 5′-TCAGTCCCTGGCTTATGGTTACC-3′ [NM_007482.3], Tgfβ1*for* 5′-TAATGGTGGACCGCAACAACG-3′, Tgfβ1*rev* 5′-TCCCGAATGTCTGACGTATTGAAG-3′ [NM_011577.1], ActivinA*for* 5′-TTCCAAGGAAGGCAGTGACCTG-3′, ActivinA*rev* 5′-GCTGCTGAAATAGACGGATGGTG-3′ [NM_008380], Mfge8*for* 5′-GGGCATCCACTGTGAAACCGAGAC-3′, Mfge8*rev* 5′-GCAATGGTGCCCCCTTCCAT-3′ [NM_008594.2], Gapdh*for* 5′-GGCATTGCTCTCAATGACAA-3′, Gapdh*rev* 5′-ATGTAGGCCATGAGGTCCAC-3′ [NM_008084].

### Tnfα and Tgfβ1 Immunohistochemistry

For the detection of Tnfα and Tgfβ1 2 days after lesion with 6-OHDA, sections from SN and CPu were washed with PBS and blocked with PBS containing 10% normal goat serum and 0.1% TritonX-100 (Roche) for 1 h at RT. Afterwards, sections were incubated with primary antibodies anti-Tnfα (sc-52746, 1:100, Santa Cruz), anti-Tgfβ1 (MAB240, R&D System, 1:50, Wiesbaden-Nordenstedt) and anti-Iba1 (1:500, Wako Chemicals) at 4°C overnight, followed by an incubation with the corresponding Cy3-conjugated secondary antibodies (goat anti-mouse Cy3 1:100, goat anti-rabbit Cy3 1:200, Cell Signaling Technologies) for 1 h at RT. Nuclei were counterstained using 4′,6-diamidino-2-phenylindole (DAPI, Roche). Fluorescence images were captured using the ZEISS AxioImager M2 imaging system (ZEISS, Göttingen, Germany).

### Quantifications of Iba1^+^ and Gfap^+^ Cells after 6-OHDA Lesion

Coronal sections from SN and CPu of 6-OHDA lesioned brains were stained with antbodies against Iba1 and Gfap (as described under “Tissue processing and immunohistochemistry” Section) and mounted onto objective slides. Immunofluorescent images were taken with a ZEISS AxioImager M2 imaging system (ZEISS, Göttingen, Germany) and were used for counting the numbers of Iba1^+^ and Gfap^+^ cells using ImageJ software (National Institutes of Health). Three animals per time-point were used, and cells in SN and CPu on control and lesioned side were counted in a 0.5 mm^2^ counting grid. Activated Iba1^+^ microglia were determined based on amoeboid morphology, thickened processes and an increased staining intensity. Numbers of cells were recalculated and presented as cells/mm^2^.

### Statistics

Data are expressed as means ± SEM. Statistical significances between multiple groups were compared using a one-way ANOVA followed by a Bonferroni’s multiple comparison test. Two-group analysis was performed using a Student’s *t*-test. Values of *p* less than or equal to 0.05 were considered as statistically significant. All statistical analyses were performed using the GraphPad Prism5 software (GraphPad Software Inc).

## Results

### 6-OHDA Injections Induce Robust Striatal Denervation and Loss of TH-positive Dopaminergic Midbrain Neurons

**Figure 1:**
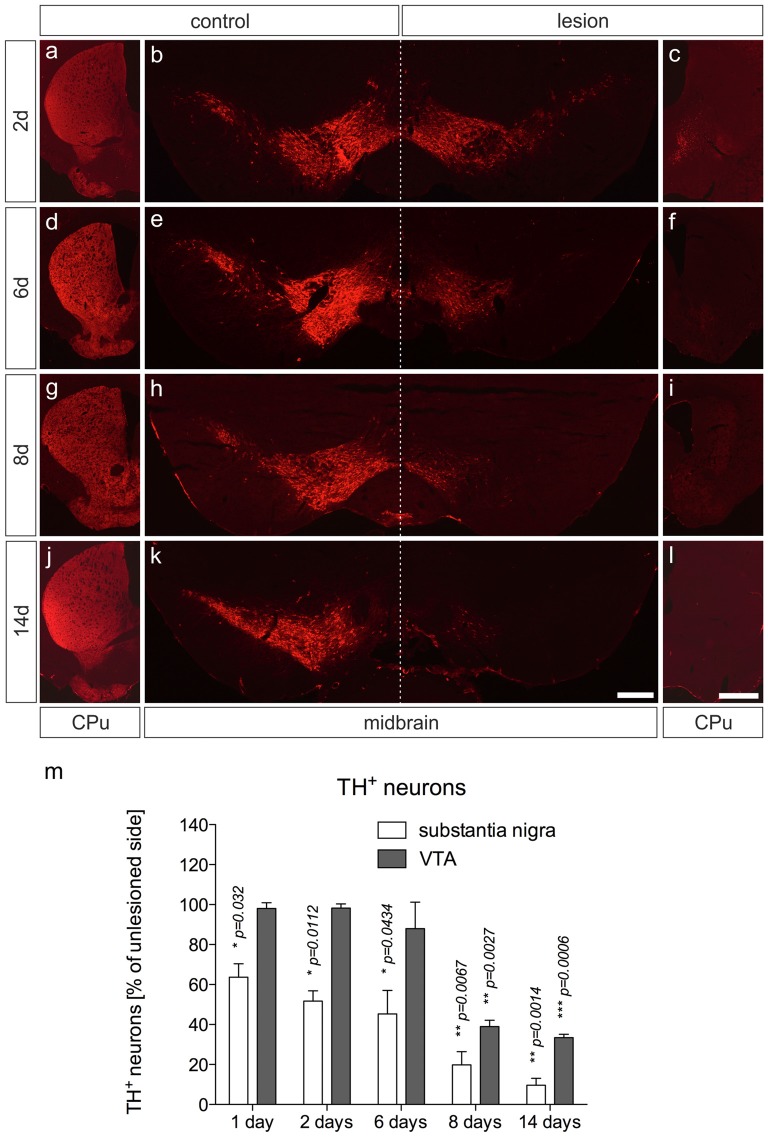
**6-OHDA injections into the MFB induce robust loss of mDA neurons and denervation in the CPu on the lesioned side.** Coronal brain sections after immunohistochemistry for TH from CPu and ventral midbrains containing SN and VTA are shown 2 days **(A–C)**, 6 days **(D–F)**, 8 days **(G–I)** and 14 days **(J–L)** after 6-OHDA injections. Whereas loss of TH^+^ fibers in the CPu occurs from 2 days onwards, substantial decrease in numbers of TH^+^ mDA neurons was evident after 6 days. Representative images from three independent experiments are shown. Scale bars indicate 300 μm **(B,E,H,K)** and 1 mm for all CPu images. Quantification of SN and VTA TH^+^ mDA neurons revealed significant decreases in SN neurons at all time-points analyzed. However, decrease of VTA neurons was delayed and reached significant differences at 8 days and 14 days **(M)**. Data are given as percentages from unlesioned sides ± SEM from three animals.

Male C57BL/6 mice were unilaterally lesioned by an injection of 5 μg 6-OHDA into the right MFB. A shown in Figures 1A–C, analysis of the nigrostriatal system 2 days after injection of 6-OHDA showed a rapid loss of TH-immunoreactivity in the CPu on the lesioned side (Figure 1C), with residual TH^+^ fiber clumps in basal parts of the CPu. The staining intensities of TH^+^ neurons in the SN were slightly decreased, and substantial neuron losses could be detected at 2 days after injection (Figures 1B,M). At 6 days (Figures 1D–F), 8 days (Figures 1G–I) and 14 days (Figures 1J–L) after injection of 6-OHDA, no TH^+^ fibers could be detected on the lesioned sides. Moreover, TH^+^ SN neuron numbers on lesioned sides gradually decreased from 2 days to 6 days (Figures 1E,M) and almost no TH^+^ SN neurons were present at 8 days (Figures 1H,M) and 14 days (Figures 1K,M) after 6-OHDA injection. Further, numbers of TH^+^ neurons in the ventral tegmental area (VTA) started to decrease from 8 days (Figures 1D,E,F,M) after 6-OHDA injections. At 14 days after injection, only some TH^+^ VTA neurons were detectable (Figures 1K,M). Together, these data clearly demonstrate that an injection of 6-OHDA into the MFB induced a rapid and robust decrease of striatal target innervation and loss of TH^+^ midbrain neurons.

### Microglia and Astroglia Reactions in the 6-OHDA-Lesioned SN

**Figure 2:**
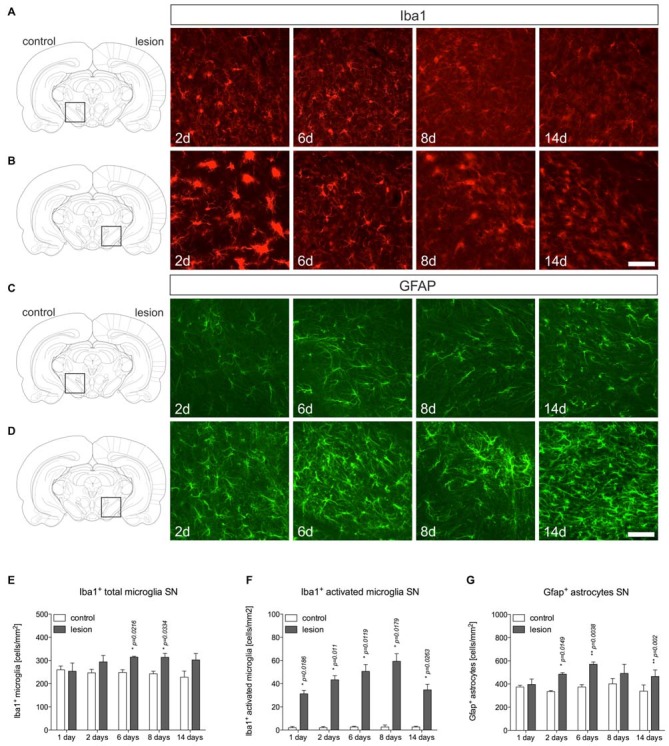
**Microglia and astroglia reactivity in the SN after 6-OHDA injections.** On the control side microglia showing ramified morphologies could be detected by Iba1-immunohistochemistry at all time-points analyzed **(A)**. Two days after 6-OHDA injections, a strong microglia reactivity was observed on the lesioned side. After 6 days, microglia start to adopt a ramified morphology indicating that microglia reactivity is rapidly inhibited **(B)**. Immunohistochemistry for Gfap demonstrated the presence of Gfap^+^ astrocytes in the SN on the control side at all time-points analyzed **(C)**. However, strong increases in Gfap-immunoreactivity and numbers of Gfap^+^ astrocytes were observed starting from 6 days after injection of 6-OHDA and persisting until 14 days **(D)**. Scale bars indicate 75 μm. Quantifications of total microglia **(E)**, activated microglia **(F)** and Gfap^+^ astrocytes **(G)** in the SN on control and lesioned sides. Data are given as numbers/mm^2^ (mean ± SEM) from three animals per time-point. *p*-values derived from student’s *t*-test are *p < 0.05 and **p < 0.01.

After establishment of the temporal pattern of midbrain TH^+^ neuron loss in male C57BL/6 mice induced by 6-OHDA, the time course of microglia and astroglia reactivity in the SN was addressed. As depicted in Figure 2, microglia on control sides showed ramified morphologies with fine processes as demonstrated using Iba1-immunohistochemistry (Figure 2A). However, 2 days after 6-OHDA injections microglia in the lesioned SN displayed reduced branching behavior, increased cell size and a stronger Iba1-immunoreactivity (Figure 2B). These microglia morphology changes have been described as reactive changes in several CNS disease models (Block et al., 2007), and are used as a crude readout for microglia activation. After 6 days, microglia morphologies started to normalize. However, even at 8 days and 14 days (Figure 2B) microglia morphologies on lesioned sides substantially differed from control sides, suggesting that microglia in the SN are rapidly and strongly reacting upon 6-OHDA lesions reaching a maximum after 2 days but seem to stay in a reactive state at all time-points analyzed. Quantifications of total microglia numbers revealed slight increases after 2 days, 6 days, 8 days and 14 days (Figure 2E). Significant increases were observed at 6 days (*p* = 0.0216) and 8 days (*p* = 0.0334). Significant increases in activated microglia numbers in the SN on lesioned sides were observed at all time-points analyzed (Figure 2F).

Using Gfap-immunohistochemistry, the reactivity of astrocytes was analyzed in midbrains on lesioned sides at different time-points. As shown in Figure 2C, Gfap^+^ astrocytes could be detected in the SN on control sides at all time-points analyzed. On lesioned sides, astrocytic staining intensities as well as total numbers of Gfap^+^ cells were increased at 2 days, 6 days, 8 days and 14 days (Figure 2D) after injection of 6-OHDA to the MFB. Quantifications of Gfap^+^ astrocytes (Figure 2G) revealed significantly increased numbers on lesioned sides after 2 days (*p* = 0.0149), 6 days (*p* = 0.038) and 14 days (*p* = 0.02).

### Microglia and Astroglia Reactions in the 6-OHDA-Lesioned CPu

**Figure 3:**
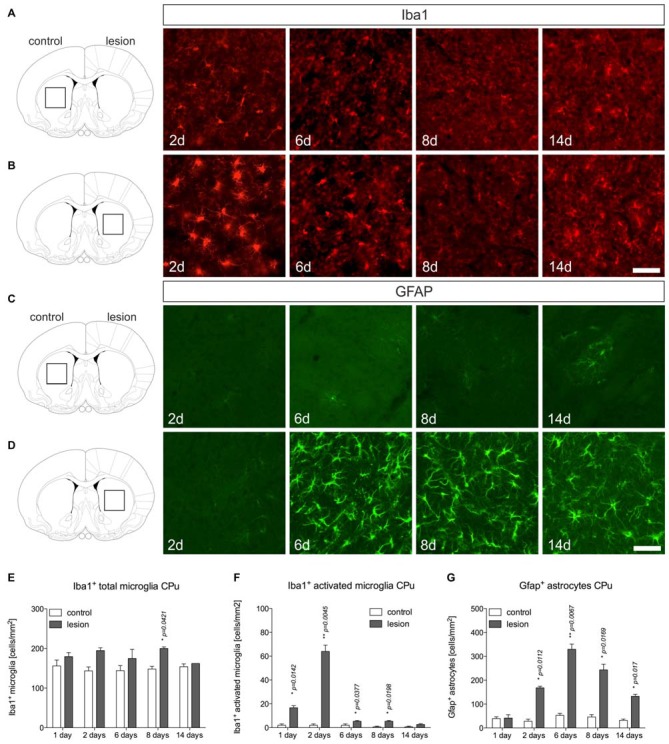
**Microglia and astroglia reactivity in the CPu after 6-OHDA injections.** Microglia showing ramified morphologies could be detected after Iba1-immunohistochemistry in the unlesioned CPu at all time-points analyzed **(A)**. Two days after 6-OHDA injections a strong microglia reactivity was observed in the lesioned CPu, which was not longer visible at 6 days, 8 days and 14 days after injection of 6-OHDA **(B)**. Immunohistochemistry for Gfap showed that virtually no Gfap^+^ astrocytes were present in the CPu on the control side at all time-points analyzed **(C)**. Comparable to the observations in the lesioned SN, strong increases in Gfap-immunoreactivity and numbers of Gfap^+^ astrocytes in the CPu were observed starting from 6 days after injection of 6-OHDA and again, persisting until 14 days **(D)**. Scale bars indicate 75 μm. Quantifications of total microglia **(E)**, activated microglia **(F)** and Gfap^+^ astrocytes **(G)** in the CPu on control and lesioned sides. Data are given as numbers/mm^2^ (mean ± SEM) from three animals per time-point. *p*-values derived from student’s *t*-test are *p < 0.05 and **p < 0.01.

Microglia and astroglia reactions were further analyzed in the 6-OHDA-lesioned CPu. Figure 3A displays microglia on control sides showing normal ramified morphologies. Two days after 6-OHDA injection, a strong microglia reactivity on the lesioned side was observed (Figure 3B). Interestingly, from 6 days onwards, ramified microglia with fine processes could be detected at 8 days and 14 days (Figure 3B). Quantifications of total microglia (Figure 3E) and activated microglia (Figure 3F) revealed slight increases in total numbers on lesioned sides at all time-points analyzed. Numbers of activated microglia reached a maximum on lesioned sides after 2 days and were only slightly different as compared to control sides at 6 days, 8 days and 14 days (Figure 3F), indicating that microglia reactivity in the CPu is rapidly downregulated after 2 days.

Analysis of Gfap^+^ astrocytes revealed virtually no Gfap^+^ cells in the CPu on control sides at all time-points analyzed (Figure 3C). After injection of 6-OHDA, astrocyte reactivity was not observed 2 days after lesion. However, at 6 days, 8 days and 14 days after 6-OHDA injection, a strong astrocytic Gfap reactivity could be detected in the lesioned CPu (Figures 3D,G). These data demonstrate that microglia reactivity in the 6-OHDA-lesioned CPu precedes astrocyte activation but is rapidly downregulated, whereas astrocyte reactivity starts from 6 days and remains activated until 14 days after lesion.

### Temporal Expression Patterns of Inflammatory Markers in the 6-OHDA-Lesioned Nigrostriatal System

**Figure 4:**
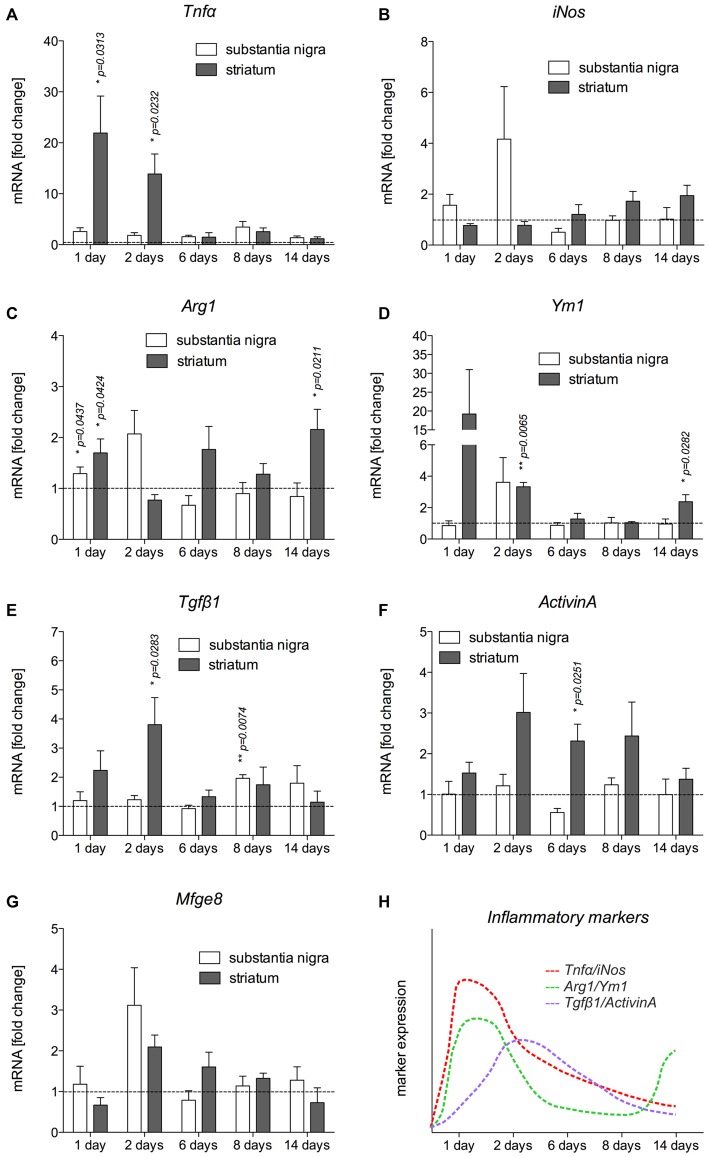
**Expression of inflammatory markers in SN and CPu after injection of 6-OHDA.** The expression levels of the M1 activation markers Tnfα **(A)** and iNos **(B)**, the M2 activation markers Ym1 **(C)** and Arg1 **(D)** as well as TGFβ1 **(E)** and ActivinA **(F)** as anti-inflammatory factors as well as* Mfge8* expression **(G)** as a TGFβ1-regulated gene have been analyzed and are displayed. Data are given as fold changes (mean ± SEM) calculated from expression levels on unlesioned control sides from at least three different animals. *p*-values derived from student’s *t*-test are *p < 0.05 and **p < 0.01. A summary of the temporal expression patterns of different markers is given **(H)**.

Based on the immunohistochemical observations of microglia and astroglia reactions after 6-OHDA injections, mRNA was isolated from the SN and CPu at different time-points after injection of 6-OHDA into the MFB to detect the expression of inflammatory markers. The expression levels of the M1 activation markers Tnfα and iNos, the M2 activation markers Ym1 and Arg1, as well as of Tgfβ1 and ActivinA as anti-inflammatory factors were analyzed. As shown in Figure 4A, *Tnfα* was significantly upregulated in the CPu of lesioned mice 1 day (*p* = 0.0313) and 2 days (*p* = 0.0232) after injection. Although a slight upregulation of Tnfα was also detectable in the SN at all time-points analyzed, these increases did not reach statistical significances. Analysis of *iNos* expression revealed that 6-OHDA lesion induced an expression of *iNos* in the SN 1 day and 2 days post lesion, whereas the expression in the CPu was not increased at these time-points. From 6 days onward, a slight increase in *iNos* expression was observed in the CPu, however, not reaching any statistical significance (Figure 4B). Ym1 and Arg1 have been recently described as markers for alternative microglia activation (Zhou et al., 2012), and thus were analyzed after injections of 6-OHDA into the MFB. *Ym1* was highly upregulated in the lesioned CPu 1 day after injection. After 2 days, increased *Ym1* expression was observed in the lesioned SN as well as in the lesioned CPu (*p* = 0.0065). At 6 days and 8 days post lesion, there were no changes in *Ym1* expression neither in the SN nor in the CPu. Interestingly, a significant upregulation of *Ym1* (*p* = 0.0282) was evident in the CPu after 14 days (Figure 4C). A similar, yet lessened regulation could further be observed for *Arg1* after lesion with 6-OHDA. As shown in Figure 4D, a significant upregulation of *Arg1* was detectable in the SN (*p* = 0.0437) and in the CPu (*p* = 0.0424) 1 day after lesion with 6-OHDA. From 2 days to 8 days after lesion, no significant changes in *Arg1* expression could be demonstrated, although increased expression was observed in the SN at 2 days and the CPu at 6 days. Noteworthy, similar to *Ym1*, the expression of *Arg1* was significantly increased (*p* = 0.0211) in the lesioned CPu after 14 days (Figure 4D). Furthermore, the expression of *Tgfβ1* and *ActivinA* as anti-inflammatory markers was also analyzed. Increases in *Tgfβ1* expression were observed after 1 day in the lesioned CPu reaching a maximum after 2 days (*p* = 0.0283). At later time-points, a tendency for increased Tgfβ1 expression in SN and CPu could be detected, however, in the SN only after 8 days a significant increase in Tgfβ1 expression (*p* = 0.0074) was noticed (Figure 4E). Analysis of *ActivinA* expression revealed a similar pattern as observed for *Tgfβ1*. Especially in the lesioned CPu, a strong increase in *ActivinA* expression was detected after 2 days, 6 days (*p* = 0.0251) and 8 days, whereas the lesioned SN showed no significant changes in *ActivinA* expression at all time-points analyzed (Figure 4F). Finally, the expression of *Mfge8* as a Tgfβ1-regulated gene that is involved in uptake of apoptotic cells was further analyzed. As shown in Figure 4G, *Mfge8* was upregulated in the SN (*p* = 0.0742) and the CPu (*p* = 0.0652) after 2 days, however, without reaching statistically significant differences. A summary of the temporal expression patterns of inflammatory and anti-inflammatory markers in the CPu, which has shown a more prominent expression of markers, is given in Figure 4H.

### Tnfα Expression is Increased in Activated Microglia in the Lesioned Nigrostriatal System

**Figure 5:**
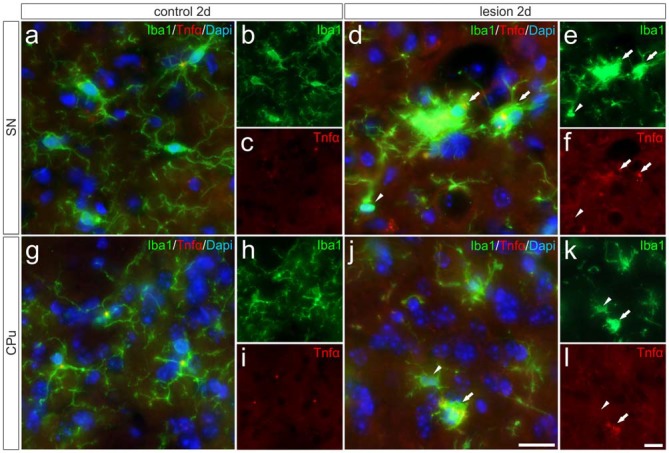
**Microglial expression of *Tnfα* in the SN und the CPu after 6-OHDA injections.** Two days after 6-OHDA-induced lesion no Tnfα expression was observed in microglia in SN **(A–C)** and CPu **(G–I)**. However, distinct Tnfα immunoreactivity could be detected on the lesioned side. Activated microglia (white arrows) showed Tnfα expression in the SN **(D–F)** as well as in the CPu **(J–L)**. No Tnfα signals were present in ramified non-activated microglia (white arrowheads) from the SN **(D–F)** and the CPu **(J–L)** on the lesioned side. Representative images from three different animals are shown. Scale bars indicate 20 μm.

In order to validate the expression of *Tnfα* analyzed on the mRNA level and to determine the cell type responsible for the increase in *Tnfα* expression, immunohistochemistry for Tnfα has been performed on brain section “Materials and Methods” days after 6-OHDA injections. Microglial *Tnfα* expression was not detectable in the SN (Figures 5A–C) and in the CPu (Figures 5G–I) on control sides. However, Figures 5D–F demonstrates that distinct Tnfα immunoreactivity was observed in activated microglia showing amoeboid morphologies on lesioned sides as indicated by white arrows. In the lesioned CPu, a similar staining pattern was observed. Again, activated microglia with typical morphologic changes (white arrows) showed positive Tnfα immunoreactivities (Figures 5J–L). In both the lesioned areas, only activated microglia as indicated with white arrows expressed *Tnfα*, whereas ramified microglia (white arrowhead) in close proximity to activated microglia showed no Tnfα immunoreactivity. These data demonstrate that activated microglia are the primary source of increased *Tnfα* expression in the 6-OHDA-lesioned SN and CPu.

### Tgfβ1 Expression is Increased in Striatal Neurons and in Activated Microglia on the Lesioned Side

**Figure 6:**
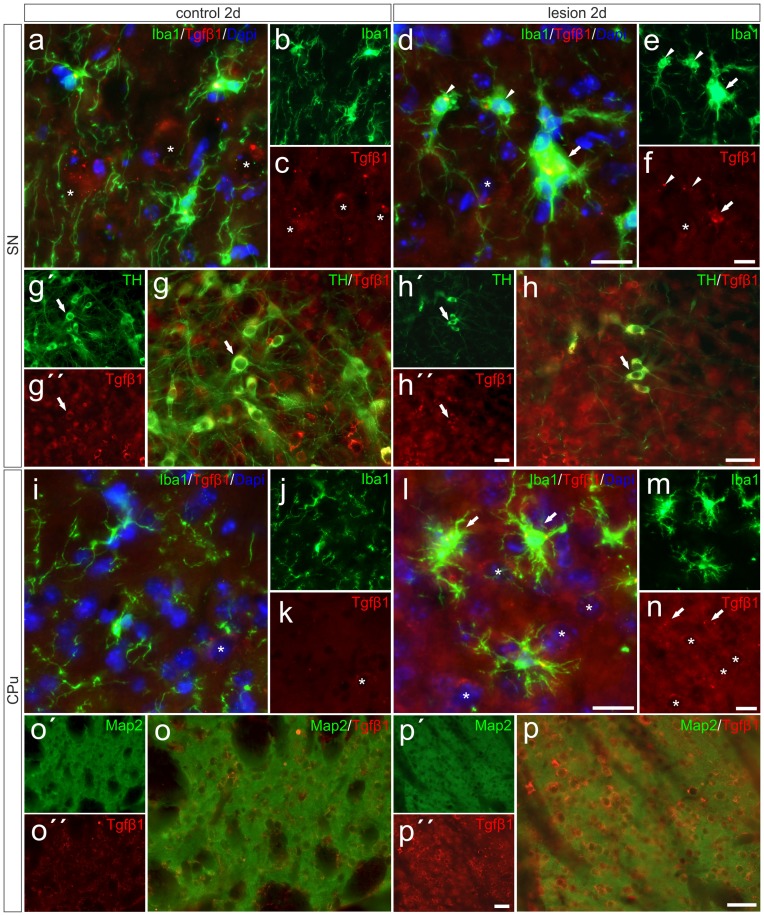
**Microglial expression of *Tgfβ1* in the SN und the CPu after 6-OHDA injections.** Two days after 6-OHDA-induced lesion weak microglial and distinct neuronal Tgfβ1 expression (white asterisks) was observed in SN **(A–C,G–G″)** and CPu **(I–K,O,O′)**. However, increased Tgfβ1 immunoreactivity could be detected on the lesioned side after 2 days **(H–H″)**. Activated microglia (white arrows) showed Tgfβ1 expression in the SN **(D–F)**, whereas ramified microglia (white arrowheads) showed no Tgfβ1 expression **(D–F)**. A strong increase in Tgfβ1 immunoreactivity was observed in the lesioned CPu 2 days after 6-OHDA injections **(L–N)**. Next to activated microglia (white arrows) and neurons (white asterisks) the neuropil displayed prominent Tgfβ1 signals in the lesioned CPu **(L,N)**. Map2-positive striatal neurons displayed a weak Tgfβ1 immunoreactivity on control sides **(O–O″)**. After lesion, strong Tgfβ1 signals could be observed in Map2-positive neurons in the CPu **(P–P″)**. Representative images from three different animals are shown. Scale bars indicate 20 μm **(A–F,I–N)** and 50 μm **(G,H,O,P)**.

After detection of increased *Tgfβ1* expression in total tissue samples from 6-OHDA-injected brains, we performed immunohistochemistry staining for Tgfβ1 at 2 days after 6-OHDA injection to elucidate its cellular localization. As shown in Figures 6A–C, *Tgfβ1* was not expressed by microglia (Iba1^+^ cells) in the SN on the control side, but by large-sized midbrain neurons showing distinct Tgfβ1 immunoreactivity (as indicated by asterisk). Figure 6G demonstrates that TH^+^ mDA neurons express Tgfβ1 (white arrows). A similar staining pattern was observed in the CPu on the control side after 2 days (Figures 6I–K). Whereas microglia show no *Tgfβ1* expression, striatal neurons displayed weak perinuclear and cytoplasmic Tgfβ1 signals (Figures 6I,K, asterisk). Figures 6D–F demonstrates that *Tgfβ1* expression was observed in midbrain neurons (asterisk) and activated microglia (white arrow) in the SN on the lesioned side. Microglia with normal ramified morphologies showed weak or no *Tgfβ1* expression (white arrowheads). TH^+^ mDA neurons further express *Tgfβ1* on the lesioned side (Figure 6H, white arrows). Interestingly, the expression of *Tgfβ1* was strongly increased in the CPu on the lesioned side (Figures 6L–N). Robust Tgfβ1 immunoreactivity was observed in striatal neurons (asterisks) and activated microglia (white arrows) whereas ramified microglia did not show expression of *Tgfβ1*. Using Map2 as neuronal maker, increased expression of *Tgfβ1* could be confirmed in striatal neurons on the lesioned side (Figures 6O,P). Moreover, an overall increase in immunoreactivity for Tgfβ1 was further detected in the neuropil and extracellular space of the CPu on the lesioned side 2 days after injection of 6-OHDA, indicating secretion and extracellular storage of Tgfβ1. These data clearly demonstrate that *Tgfβ1* expression is increased in striatal neurons and in activated microglia in the CPu on the lesioned side 2 days after injection of 6-OHDA.

## Discussion

In the present study, we have demonstrated that microglia quickly adopt a reactive state in the SN and CPu after injection of 6-OHDA into the MFB. This microglial activation precedes the substantial loss of TH^+^ neurons in the midbrain. The rapid microglial response is followed by an astrocytic reaction, which persists until 14 days after injection whereas microglial reactivity decreased and morphological changes of microglia normalize from 6 days onwards. The temporal microglia activation pattern correlated with the expression of the pro-inflammatory markers *Tnfα* and *iNos*, the latter of which being the essential enzyme for NO production. Both factors have been shown to be involved in driving the progression of neurodegeneration (Block et al., 2007). Using immunohistochemistry, we were able to demonstrate that activated microglia in the SN and CPu are the source of *Tnfα* expression 2 days after injection of 6-OHDA into the MFB. This microglia activation in the 6-OHDA model of PD, which precedes degeneration of mDA neurons has been demonstrated in several recent studies using different 6-OHDA injection approaches (Henry et al., 2009; Marinova-Mutafchieva et al., 2009; Walsh et al., 2011; Stott and Barker, 2014), and underline the importance of microglial activation in driving the progression of mDA neurodegeneration. Indeed, inhibition of microglia reactions either by the anti-inflammatory CX3CL1 (Pabon et al., 2011), or by application of doxycycline (Lazzarini et al., 2013) resulted in reduced microglia-mediated neuroinflammation and an increased survival of mDA neurons after 6-OHDA injections. These results indicate the importance of attenuating microglia activation in order to prevent excessive neurodegeneration. Tgfβ1 is one of the endogenous factors that have been shown to be involved in regulation of microglial activation (Spittau et al., 2013; Butovsky et al., 2014). *Tgfβ1* expression has been described to be low under normal conditions and to increase in the lesioned CNS with microglia and/or macrophages being the major sources (Lehrmann et al., 1998; Vincze et al., 2010; Pál et al., 2012). Here, we describe that *Tgfβ1* was upregulated predominantly in the CPu, reaching a peak after 2 days. Although we were able to show that neurons predominantly express *Tgfβ1* on control and lesioned sides, activated microglia show increased *Tgfβ1* expression in the lesioned SN as well as in the lesioned CPu. The increased *Tgfβ1* expression followed the expression peak of the pro-inflammatory markers *Tnfα* and *iNos*, indicating that Tgfβ1 might be involved in deactivation of microglia, which might subsequently result in reduced microglia-mediated neurodegeneration. The molecular mechanisms by which Tgfβ1 promotes its anti-inflammatory effects on microglia are only partially understood. A possible mechanism might be the Tgfβ1-dependent induction of NG2^+^ microglia as shown after LPS injection into the CNS (Xiang et al., 2015). Interestingly, the induction of NG2^+^ cells has also been reported after 6-OHDA injections (Kitamura et al., 2010). NG2 is a member of the CSPG (chondroitin sulfate proteoglycan) superfamily and has been shown to be a substantial part of the glial scar (Tan et al., 2005). Moreover, recent studies suggest that NG2 expression in microglia alters their functional properties. Gao et al. (2010) demonstrated that NG2 is involved in regulating the production and secretion of inflammatory cytokines in activated microglia. In addition, Smirkin et al. (2010) observed that NG2^+^ macrophages promote neuron survival in ischemic lesions by increased secretion of neuroprotective factors such as GDNF and IGF-1. Release of neurotrophic factors, such as GDNF, has been further reported in activated (Gfap^+^) astrocytes, extending the functions of reactive astrocytes beyond their well-established role during glial scar formation (Chen et al., 2005). The observed astrocyte activation in the present study might also be involved in the attempt to support neuron survival and a functional recovery after 6-OHDA injections. The observed temporal activation pattern suggests, that microglia might be involved in driving astrocyte reactions, which has also been proposed for several pathologies in the CNS (Liu et al., 2011). However, the molecular mechanisms underlying microglia-astroglia communication are not well understood and need to be further elucidated.

The temporal and spatial expression patterns of *Tgfβ1*, which we demonstrate in the present study, suggest that *Tgfβ1* might be upregulated in order to decrease microglial activation and further change their functional features. Interestingly, we were able to detect increased expression of markers for alternative (M2) microglial activation after injection of 6-OHDA into the MFB. Expression levels of *Ym1* and *Arg1* increased at early time-points (1 day, 2 days) after lesion, with a second peak in the CPu after 14 days. Increased expression of *Tgfβ1* preceded the upregulation of *Ym1* and *Arg1* after 14 days. As we have recently demonstrated, microglial Tgfβ secretion and autocrine activation of Tgfβ signaling are essential for IL-4-mediated upregulation of Ym1 and Arg1 (Zhou et al., 2012). It should be considered that Tgfβ1 might serve as an important endogenous factor that regulates different microglia activation states, inhibiting M1 activation and shifting microglia phenotypes towards M2 activation. However, these Tgfβ1-mediated mechanisms, which have been shown *in vitro* (Zhou et al., 2012; Spittau et al., 2013), must not necessarily be relevant *in vivo* and at this stage remains speculative.

It is noteworthy that the expression patterns of *ActivinA* were similar to that of *Tgfβ1.* ActivinA has also been shown to exert anti-inflammatory effects on activated microglia (Ogawa et al., 2006; Sugama et al., 2007). Moreover, we have demonstrated that the expression of *Mfge8* was increased after 2 days in the SN and to a lower extent in the CPu. Although these increases in *Mfge8* expression did not reach significant differences, this secreted factor might be involved in binding to apoptotic neurons to initiate a subsequent uptake by microglia. Interestingly, we have previously shown that Tgfβ1 increases the engulfment of apoptotic cells by microglia, by increasing microglial *Mfge8* expression (Spittau et al., 2015).

The present study suggests that Tgfβ1 might be involved in regulating and shaping microglia activation states in the 6-OHDA mouse model of PD. Current treatment strategies are focussed on restoring the dopamine deficits by application of L-3,4-dihydroxyphenylalanine (L-DOPA) and thereby reducing motor symptoms of PD (Barbeau, 1969). However, L-DOPA itself has been reported to induce glia reactions (Bortolanza et al., 2014) and thus, is not an optimal treatment option. The anti-inflammatory properties of Tgfβ1 and other immunoregulatory factors should be considered during establishment and validation of treatment paradigms in animal models of PD that aim to reduce neurodegeneration. In the 6-OHDA model, the infusion of GDNF and Tgfβ1 is more protective compared to the treatment with GDNF alone (Gonzalez-Aparicio et al., 2010). Similar results have been obtained in the MPTP mouse model of PD (Schober et al., 2007). The combination of GDNF—as a very potent neurotrophic factor—and Tgfβ1, as an anti-inflammatory factor which reduces microglia-mediated neuroinflammation, could be a powerful therapeutic option, especially, due the lack of GDNF to inhibit the activation of mouse microglia, which do not express essential signaling receptors (Ku et al., 2013; Zlotnik and Spittau, 2014).

However, the molecular mechanisms of how Tgfβ1 affects microglia activation states and functional properties are still only partially understood. Moreover, excessive Tgfβ1 has detrimental effects on CNS homeostasis and neuron survival (Wyss-Coray et al., 1995, 1997) and therefore, detailed knowledge about cellular expression, release, extracellular storage and activation of Tgfβ1 is necessary before considering application of Tgfβ1 as a therapeutic agent. Furthermore, it remains to be elucidated whether neuron- and/or microglia-derived Tgfβ1 is important to silence microglia activation, thereby reducing neurodegeneration in the 6-OHDA mouse model of PD. In conclusion, the data presented in this study demonstrate that microglial activation precedes the delayed astrocytic response and further reveal the temporal and spatial expression patterns of inflammatory and anti-inflammatory factors after an unilateral 6-OHDA injection. The expression patterns of Tgfβ1 and ActivinA suggest that these factors might be involved in regulating microglial activation in this toxin-based model of PD. However, further *in vivo* studies have to be performed in order to draw conclusions about the mechanisms of Tgfβ1 and microglial Tgfβ signaling in the regulation of microglia functions in PD models.

## Author Contributions

All authors listed, have made substantial, direct and intellectual contribution to the work, and approved it for publication.

## Conflict of Interest Statement

The authors declare that the research was conducted in the absence of any commercial or financial relationships that could be construed as a potential conflict of interest.
